# Housing developers’ perceived barriers to implementing municipal sustainability requirements in Swedish sustainability-profiled districts

**DOI:** 10.1007/s10901-021-09923-z

**Published:** 2021-11-24

**Authors:** Melissa Candel, Niklas Törnå

**Affiliations:** 1 Division of Real Estate Planning and Land Law, Department of Real Estate and Construction Management, KTH Royal Institute of Technology, Teknikringen 10B, 100 44 Stockholm, Sweden; 2 Division of Industrialized and Sustainable Construction, Department of Civil, Environmental and Natural Resources Engineering, Luleå University of Technology, Luleå, Sweden

**Keywords:** Housing developers, Sustainability requirements, Implementation barriers, Sustainable urban development, Public land development

## Abstract

Swedish municipalities are taking an active role in shaping and implementing sustainability-related policies in urban development by initiating and governing sustainability-profiled district developments on municipally owned land. To drive sustainable development and innovation in these districts and develop future policies, municipalities use land allocation agreements to set project-specific sustainability requirements on housing development projects that go beyond the current national building regulations. Developers play a key role in implementing these municipal sustainability requirements. The purpose of this paper is to explore housing developers’ perceived barriers to implementing municipal sustainability requirements in their projects, which ultimately constrain possibilities for municipalities to drive sustainable development. Findings are based on case studies of two sustainability-profiled district developments in different Swedish municipalities. Main barriers perceived by the developers could be categorized into: (1) increased costs when adapting to unforeseen changes that constrain project budgets and (2) conflicting interests and objectives between interdependent actors. These barriers are contextualised within the relationship between the developers and municipalities. Contributions are made to literature on developers’ roles and perspectives in sustainability-oriented urban development. We illustrate how conflicting short-term and long-term interests between developers and municipalities complicate and impede problem solving in housing development projects. This calls for more research on these actors’ interests, and how they align and conflict in these types of projects. Findings also illustrate how developers resolve issues through interactions with municipalities, indicating collaborative problem solving processes to investigate further.

## Introduction

Contemporary sustainable housing development practices are changing the roles of public and private actors (Hagbert & Malmqvist, 2019). It is becoming increasingly common for Swedish municipalities to initiate and govern the development of high-profile residential districts that are designated to act as models for sustainable urban development and testbeds for innovation (see e.g. Storbjörk & Hjerpe, 2014; Hagbert & Femenias, 2016; Kågström, 2020), here referred to as sustainability-profiled districts. While municipalities in Sweden have a monopoly on urban planning and land use, as is the case in several countries (Tambach & Visscher, 2012), they also use their position as landowners to govern sustainability in local urban development (Francart et al., 2019). Similar utilisation of public land has been observed in the Netherlands, Finland, Denmark, Switzerland and Germany (Bulkeley & Kern, 2006; Greber, 2016; Smedby & Quitzau, 2016; Tambach & Visscher, 2012; Valtonen et al., 2018). In Sweden, municipalities use land allocation agreements to place project-specific sustainability requirements on building development projects that exceed the current building regulations (Brokking et al., 2020; Caesar, 2016; Candel et al., 2021; Francart et al., 2019; Smedby, 2016; Smedby & Quitzau, 2016; Storbjörk et al., 2018), referred to here as municipal sustainability requirements. This practice is particularly common in sustainability-profiled district developments.

Property developers play a key role in implementing sustainability-related policies in urban development, although they do not receive much attention in this literature (Storbjörk et al., 2018; Taylor et al., 2012). On the other hand, there is a general recognition of the potential that collaborations between public and private actors in housing have for contributing to sustainable development in cities (Fell & Mattsson, 2021). In Sweden, developers are defined as “those who carry out, or assign others to carry out, design, construction, demolition or groundworks for their own account” (SFS, 2010:900). Findings from previous studies suggest that different types of property developers perceive different challenges specifically related to project-specific municipal sustainability requirements (Candel et al., 2021; Hagbert & Malmqvist, 2019; Storbjörk et al., 2018). Here we focus specifically on housing developers, referring to a type of property developer that finances and develops residential buildings that they either intend to sell or rent out, which can also be termed residential property developers. Since housing developers are responsible for interpreting and implementing municipal sustainability requirements in their projects and thereby realising municipalities’ sustainability objectives in sustainability-profiled districts, the specific challenges that they perceive warrant further investigation.

There is a significant body of literature, mainly from the construction management field, that identifies developers’ perceived barriers to sustainable construction (SC) (e.g. Deng & Wu, 2014; Häkkinen & Belloni, 2011; Isaksson & Linderoth, 2018; Zainul Abidin et al., 2013), also referred to as sustainable building, and to innovation and change more generally (e.g. Ivory, 2005; Vennström & Eriksson, 2010). These barriers provide an overview of the challenges that developers face with regards to implementing new SC solutions and practices within their individual projects. However, it is not certain how these perceived barriers to SC translate to the implementation of municipal sustainability requirements where there is a contractual relationship with a municipality involved. Here we explore these perceived barriers to SC further in this particular context, building on the assumption that sustainability-profiled district developments initiated and governed by municipalities form distinct project environments for housing developers to navigate. The overall purpose of the paper is to explore how housing developers’ perceived barriers to SC influence their implementation of municipal sustainability requirements in Swedish sustainability-profiled districts. We answer Storbjörk et al.’s (2018) call for more research exploring the perspectives of property developers in sustainability-oriented urban development and contribute to the growing discussion on the topic (e.g. Taylor et al., 2012; Hagbert & Femenias, 2016; Hagbert & Malmqvist, 2019; Brokking et al., 2020; Kågström, 2020).

The rest of the paper is structured as follows. In the following section we present a description of the Swedish planning context to elaborate on what is meant by municipal sustainability requirements and how they fit into the planning process. This is followed by previous literature on developers’ perceived barriers to SC and to innovation and change more generally. Here we also present principal-agent theory, which provides the conceptual framework for analysing the relationship between developers and municipalities in this context. After this we present two case studies that were carried out, data collection methods and the analysis. This is followed by a presentation of the results, which are then discussed in relation to the previous literature. The paper is concluded with the main theoretical contributions, implications for policy and practitioners, the limitations of the study and suggestions for future research.

## Municipal sustainability requirements in the Swedish context

In Sweden, municipalities have what is often referred to as a monopoly on planning in their geographical area, which entitles them to decide when, where and how land development takes place (Blücher, 2013). In their role as public authorities, Swedish municipalities have two legally binding formal planning instruments which are intended to regulate urban development and steer it in a sustainable direction (Hedström & Lundström, 2013). These are detailed development plans (detaljplaner) and building permits (bygglov), produced in that order (ibid). In the detailed development plans, municipalities can specify permitted uses of buildings, the size of buildings and the size of individual dwellings (Valtonen et al., 2018). It has become a common practice in Sweden to have housing developers carry out the early phases of their projects in parallel with the detailed planning process and participate in preparing detailed development plans (Kalbro, 2013). This is supposed to give housing developers the opportunity to influence the detailed planning process, improve coordination between planning and construction (Caesar, 2016; Kalbro & Lindgren, 2018) and accelerate the planning process (Bröchner et al., 2020). However, the planning process in Sweden is to a large degree controlled by a municipality’s traditions (Olander & Landin, 2008), and many still choose to allocate land after the detailed planning process. In their study, Olander and Landin (2008) found that housing developers perceive the Swedish planning process for new developments to be time-consuming, costly, unfair, and a source of uncertainty.

In addition to their role as a public authority, municipalities in Sweden have other options to steer urban development in their role as land owners (Francart et al., 2019). Housing developers consider Swedish municipalities important suppliers of buildable land since many of them own significant portions of the land in their geographical area (Caesar, 2016). In the case of public land development in Sweden, housing developers are assigned to municipal land, either during or after the detailed planning process, by signing land allocation agreements. Land allocation agreements are defined as “an agreement between a municipality and a developer [byggherre] that gives the developer the sole right to negotiate with the municipality for a limited time and under given conditions on the transfer or lease of a certain piece of land owned by the municipality for the purpose of development” (SFS, 2010: 900). Housing developers then work with the municipality in what Caesar (2016) describes as an ‘interdependency-based partnership’ to produce a final development agreement. After this the serviced building plots are transferred to the developers, building permits are issued and building development can begin (Francart et al., 2019). In efforts to drive sustainable development, many municipalities in Sweden use sustainability criteria when choosing developers to allocate land to and include project-specific sustainability requirements that go beyond the national building regulations in the land allocation agreements, typically included as attachments in a separate document (Caesar et al., 2013; Brokking et al., 2020; Candel et al., 2021).

There is some controversy regarding these types of municipal requirements (see Svensson & Torbäck, 2016). In 2014, a new law was introduced in Sweden that prohibits municipalities from setting requirements on technical properties that go beyond those in the national building regulations (Boverkets byggregler), also termed special requirements, in connection to implementing detailed development plans (SFS, 2010: 900). However, in their role as land owners, municipalities still set additional requirements, and the law is not considered as clear on this matter (Francart et al., 2019). Either way, Högström et al (2019) assert that translating sustainability goals into specific requirements during planning is an important part of the process that links individual development projects to municipalities’ strategies. Francart et al. (2019) have explored the use of municipal sustainability requirements, and to some extent the relationship between municipalities and developers, from the municipalities’ perspective. In order to understand how these requirements are implemented in the housing development projects, a more in-depth investigation of the housing developers’ perspectives is needed.

## Previous literature on developers and theoretical framework

### Developers’ perceived barriers to sustainable construction

We adopt a specific focus on SC because this is generally also the focus of municipal sustainability requirements. By reviewing previous literature on developers’ perceived barriers to implementing SC we identify two main categories; increased financial risk and conflicting objectives.

#### Increased financial risk

Developers’ understanding is crucial for the development of SC, which is ultimately determined by their interest in and willingness to adopt SC considerations in their projects (Häkkinen & Belloni, 2011; Isaksson & Linderoth, 2018). A lack of developer understanding and competence is therefore a major barrier to SC (ibid). For housing developers, the fear of increased costs appears to be one of the most significant barriers to the consideration of SC (Opoku & Ahmed, 2014; Osmani & O'Reilly, 2009; Shen et al., 2017; Williams & Dair, 2007; Zainul Abidin et al., 2013). A big issue here is the way that risks and costs are distributed among actors. For instance, numerous studies point to a lack of benefits and incentives for housing developers to adopt green building practices (e.g. van Bueren & Primeus, 2002; Circo, 2008; Deng & Wu, 2014; Isaksson & Linderoth, 2018). Increased costs for solutions that lack benefits simply reduce developers’ potential profits, meaning SC may be perceived as an unrewarded financial risk.

Life cycle studies on green buildings recognize that savings accrued throughout the buildings life cycle might outweigh additional costs from the construction phase (Circo, 2008). However, lower operational and maintenance costs in the long-term for the end users are difficult for housing developers to predict and therefore to profit from (van Bueren & Primeus, 2002; Circo, 2008). End users are often not involved in decision-making processes and housing developers are instead “strongly fixated on the investment decisions related to the development costs” which often sways them away from SC practices (van Bueren & Primeus, 2002; 82). In addition to this, developers lack sufficient information regarding the costs of implementing various sustainable solutions and practices, and they are concerned about the reliability of new technologies (Williams & Dair, 2007; Osmani & O’Reilly, 2009). Difficulties in procuring green products locally and lacking local technical skills can also make it difficult for developers to adopt new technologies from their local industry, which results in increased costs for importing products (Zainul Abidin et al., 2013). Furthermore, Häkkinen and Belloni (2011) found that they perceive a lack of support and clear methods for both setting SC goals and interpreting them as requirements and criteria for procurement. Developing this know-how requires resources, thereby further increasing the costs and associated risks of SC.

#### Conflicting objectives

SC is not always aligned well with developers’ other interests and objectives. For housing developers, SC lacks a sense of urgency due to a lack of customer demand and government regulations (Osmani & O'Reilly, 2009; Zainul Abidin et al., 2013). Governmental policies and regulations are typically considered as one of the main drivers and enablers for SC (Olanipekun et al., 2016; Zainul Abidin et al., 2013), but the wrong type of regulation or a lack of regulation can act as a barrier (Häkkinen & Belloni, 2011). Toppinen et al. (2018) also point out that regulations that drive sustainability reflect societal needs rather than consumer needs, which developers are more concerned with. A perceived lack of customer demand is a major concern for most developers since it suggests that SC will not increase the value of their final product (Häkkinen & Belloni, 2011; Zainul Abidin et al., 2013). Seeking to develop their SC capabilities in the long-term is thereby in conflict with catering to their customers to increase the value of their final product in the short-term. However, Osmani and O'Reilly (2009) found that this is not necessarily the case for developers operating in niche markets. It should be noted that developers are more likely to consider SC solutions when constructing buildings that they will be operating, such as offices, due to long-term benefits like lower energy and maintenance costs (Zainul Abidin et al., 2013). Developers’ overall objective to increase the value of their final product is also connected to their desire to minimise costs, discussed previously.

Sustainable development also covers a very wide range of environmental, social and economic sustainability issues. Sustainability objectives in housing development can thereby differ significantly and are often in conflict with each other (Williams & Dair, 2007), seen to be the case in sustainability-profiled district developments as well (Francart et al., 2019). In their study, Williams and Dair (2007) found that conflicting sustainability objectives sometimes force developers to prioritise one objective over another. This is done by determining which sustainability objective is more desirable given the developers’ priorities and local circumstances (ibid). The majority of the previous literature on developers’ perceived barriers to SC is solely concerned with environmental sustainability meaning social and economic sustainability do not receive as much attention, which is a common critique of sustainable urban development (Martin et al., 2018). This suggests that important conflicts between different types of sustainability objectives have been overlooked in much of this previous literature.

This previous literature is largely focused on explaining why developers choose not to implement SC in their projects and less on the actual implementation process. For this reason, the studies are mainly concerned with developers’ initial consideration of SC, which involves weighing benefits against risks and costs to evaluate the business case for, or against, it. However, previous literature on developers’ perceived barriers to innovation illustrate how implementation processes pose their own set of challenges, discussed further in the next section. How perceived barriers emerge and develop during the process of implementing new SC solutions and practices, ergo after a developer has already decided they will attempt to implement them, thus warrants further investigation. Here we explore the perceived barriers to SC as potential barriers to implementing municipal sustainability requirements, paying particular attention to their implications during the actual implementation process. Previous research has also focused on more typical developments. Here we contribute with an exploration of developers’ perceived barriers to SC in flagship projects with high sustainability ambitions imposed by a local authority. The objective is to generate more in-depth and context-dependent knowledge of how these barriers are perceived and what consequences they have for implementing municipal sustainability requirements in Swedish sustainability-profiled districts. Exploring how barriers are perceived in these types of projects could generate valuable insights for improving the sustainable value generated by them and contribute to ongoing discussions about sustainability issues in urban development and the housing market at large. We do this by the following research question:

##### RQ1

How are barriers to SC perceived by housing developers in relation to implementing municipal sustainability requirements in sustainability-profiled districts?

### The role of developers and their project relationships in innovation

Sustainable development calls for innovation and change. It has long been recognised that developers, which are more commonly referred to as construction clients in the construction management field, play an important role in the process of both initiating and implementing innovation in development projects (Kulatunga et al., 2011; Lindblad & Karrbom Gustavsson, 2021). Developers are often presented as important change agents and innovation champions because of their position to govern projects by setting specifications and formulating requirements during procurement (e.g. Havenvid et al., 2016; Kulatunga et al., 2011). However, this is also challenged due to the numerous institutional, industrial and attitudinal barriers to change that they perceive (Vennström & Eriksson, 2010). Perceived institutional barriers include government regulations and formal standardized contracts. The industry culture, which is typically described as conservative, also results in a strong resistance to any deviations made from the established industry practices (ibid). Previous studies have found that developers tend to perceive innovation as too risky and not profitable enough to implement in their projects (e.g. Davies et al., 2014; Häkkinen & Belloni, 2011; Ivory, 2005), which aligns with the previous research on their perceived barriers to SC. As a project-based industry, developers tend to focus on projects rather than processes, which results in a short-term focus on costs and productivity that hinders learning and innovation (Häkkinen & Belloni, 2011). Furthermore, since innovations in construction are usually implemented in projects, rather than within the firm, the implementation process typically involves complex negotiations with other actors in the project (Harty, 2008). The process of implementing innovation in projects is thus heavily influenced by relationships between actors within the project.

Relationships between projects and other actors in the project environment can also impact project processes and outcomes and generate varying degrees of uncertainty (Jensen et al., 2006). Olander and Landin (2005) have previously identified housing developers and municipalities as two of the major stakeholders during the earlier phases of housing development projects. However, there is a lack of research that investigates their relationship from the perspective of the developers in the context of implementing innovation. Municipal sustainability requirements, which typically entail innovation, are formulated by municipalities and implemented in housing development projects. This means that negotiations over their implementation are not only held between actors within the housing development projects, but also between the municipalities and housing developers (c.f. Candel et al., 2021). The relationships between municipalities and housing developers would thus likely affect the ways in which housing developers choose to, or are able to, address perceived barriers as they emerge, making them especially significant for understanding the particular context of Swedish sustainability-profiled districts. Here we address the influence of the relationship between housing developers and municipalities on the process of implementing innovation during early project phases. These relationships are interpreted here using principal-agent theory, presented in the following section, and their implications for addressing perceived barriers are explored in order to answer the second research question:

#### RQ2

How are perceived barriers to implementing municipal sustainability requirements influenced by housing developers’ relationships with municipalities?

### Principal-agent theory

In the context of projects, principal-agent theory has been applied to a variety of project relationships (see e.g. Turner & Müller, 2004; Jensen et al., 2006; Parker et al., 2018). An agency relationship is defined as one party (the principal) depending on another party (the agent) to perform a service or undertake some kind of action on their behalf (Turner & Müller, 2004). In other words, the principal delegates work to the agent (Eisenhardt, 1989a). Principal-agent theory is adopted here since it is considered particularly appropriate for the study of hierarchical and contractual relationships (Jensen et al., 2006), such as the relationship seen between municipalities and housing developers in Swedish sustainability-profiled districts. Principal-agent theory is also considered suitable for studying conflicting interests and objectives between actors in agency relationships (Shapiro, 2005), which aligns with the second barrier to SC being explored here. The theory is based on the assumption that there is a misalignment between the principal and agent’s objectives and interests, and posits that aligning them through the use of incentives is prevented by asymmetric information and knowledge (Eisenhardt, 1989a; Hart & Holmström, 1987; Waterman & Meier, 1998).

The information advantage that agents typically have over principals, who are not able to monitor everything that the agent does, can lead to a lack of effort and opportunistic behaviour, referred to as moral hazard (Eisenhardt, 1989a). Principals protect themselves against moral hazard by monitoring the agent’s work, enforcing contracts and choosing appropriate agents (Jensen et al., 2006). Although, there remains the problem of agents that misrepresent their skills and abilities when being hired, which is referred to as adverse selection (Eisenhardt, 1989a). However, from the perspective of the agent, the relationship is arguably a lot more complex. There are problems inherent in serving multiple principals with competing and possibly conflicting interests, which is often the situation that agents find themselves in (Shapiro, 2005). While principals are competing with each other for influence over the agents, the agents are forced to weigh their different interests with their own self-interest and make compromises and trade-offs between them (Waterman & Meier, 1998).

In principal-agent theory, a distinction is often made between behaviour-based contracts and outcome-based contracts. Behaviour-based contracts, such as employment contracts, typically require more monitoring, while outcome-based contracts require the measurement of outcomes and allocate more risk to agents (Eisenhardt, 1989a). Outcome-based contracts are based on the agent’s achievement of specific performance standards and requirements. Municipal land allocation agreements can thereby be considered outcome-based contracts. The typical challenges with these types of contracts are related to difficulties in gathering information on various outcomes to be able to measure performance and allocating risk to agents who are commonly considered to be more risk averse (Eisenhardt, 1989a; Jensen et al., 2006). This shift of risks from the principal to the agent is a result of outcome uncertainty. Outcomes are not completely dependent on the agent’s effort and work because they can also be affected by various environmental factors (Shapiro, 2005). Here we explore these challenges further in the case of municipal land allocation agreements with municipal sustainability requirements.

## Research design

### Case studies

A comparative case study of two ongoing sustainability-profiled urban district developments in Sweden is used to develop in-depth and context-dependent knowledge of housing developers’ perceived barriers to implementing municipal sustainability requirements (Flyvbjerg, 2006). Using multiple case studies enabled us to make comparisons and improve the validity of the results. An abductive approach was used, meaning the empirical fieldwork and case analysis evolved simultaneously while consulting previous literature iteratively (Dubois & Gadde, 2002). This approach is suitable for case study research where the objective is to create new knowledge rather than confirming existing theories (Eisenhardt, 1989b). The urban district developments Stockholm Royal Seaport (SRS) and Nature Town (NT) were chosen due to the municipalities’ high ambitions regarding sustainable development and their symbolic value as flagship projects. As a consequence, the municipalities in both cases place many high sustainability requirements on the housing development projects to challenge the developers to develop and implement SC solutions and practices. The municipalities put more resources into these projects than their typical projects as they are expected to create more societal value in the long-term in the form of these new sustainable practices and solutions. These are extreme and somewhat unusual cases, which can reveal aspects that have been overlooked in the study of more typical cases (Stake, 1995). Flyvbjerg (2006; 229) argues that extreme cases also generate more information about phenomena as they “activate more actors and more basic mechanisms”, which makes it possible for researchers to explore deep rooted sources of problems. By studying these particular cases we expect to see perceived barriers differ somewhat from the previous literature, either in their nature and/or in scope.

SRS and NT are both large, long-running and complex urban district developments in Sweden. The former was initiated and is governed by Stockholm municipality and the latter by Luleå municipality. Stockholm is located in central Sweden and Luleå in the northern part of the country, both on the eastern coast. Both district developments are divided into different stages that are mostly implemented sequentially, with some stages running in parallel. Each stage has a different set of municipal sustainability requirements and acts as an experiment for the municipalities to develop ways of governing and advance the development and adoption of innovative SC solutions and practices. In other words, the municipal sustainability requirements frame the public value that the municipalities seek to create. One of these stages from each district development was studied, which made it possible to investigate multiple housing developers’ implementation of the same municipal sustainability requirements.

The stage that was studied in SRS consists of eleven private housing developers all building residential condominiums, and the stage in NT consists of twelve private housing developers building residential condominiums, some of which changed to rental apartment buildings during the planning process. The study was carried out during the early phases of the housing developers’ projects after land allocation agreements had been signed. The early phase of a construction project is considered important for innovative activities and planning project execution because important conceptual decisions and strategic choices are made and technical solutions that satisfy the project requirements are being developed (Kolltveit & Grønhaug, 2004). During this time the housing developers conduct pre-studies, work on developing their solutions and design to meet both the municipalities’ requirements and their own. They also start planning the production and coordinate their plans and activities with the municipality and neighbouring developers. A notable difference between the two cases in this study regards the housing developers’ participation during the detailed planning process. In SRS, the early phase of the housing development projects are carried out in parallel with the detailed planning process. In NT, on the other hand, the detailed development planning process is finalized prior to the housing developers signing land allocation agreements with the municipality, meaning they had finished detailed development plans to plan their projects around from the start.

### Data collection and analysis

Semi-structured interviews were the main method used to explore the housing developers’ perceived barriers to implementing municipal sustainability requirements in the two cases. The analysis and results are therefore largely focused on the material gathered from these interviews with the developers. Interview questions included background information and themes such as their working process, perceived challenges, the municipal sustainability requirements and working with other actors including the municipality. To gain more general background knowledge of the two cases, semi-structured interviews with municipal representatives, including two focus group discussions in NT, were conducted along with a review of documents. This material was used to gain a general understanding of how the municipalities govern the housing developers, what municipal sustainability requirements are included in the stages that were studied, important events and decisions that had been made and the overall history of the districts. Non-participant observations from different types of meetings between the housing developers and the municipality in SRS were also conducted to see first-hand how these actors discussed perceived barriers with each other. The empirical material, which is summarised in Table 1, was collected between March 2018 and May 2019 in SRS by one of the researchers, and between May 2019 and June 2020 in NT by the other researcher. All of the interviews and focus group discussions were recorded and transcribed by the researchers.

**Table 1:** Empirical material used in the study

Case	Empirical material
Stockholm Royal Seaport (SRS)	*Interviews (60–120 min/interviewee)*:
	11 private housing developer project managers, all building condominiums (HD SRS)
	1 office developer project manager
	1 construction consultant project manager
	3 municipal planning project managers (2 were interviewed twice) (M SRS)
	1 municipal sustainability strategist
	1 municipal consultant responsible for sustainability coordination
	1 municipal contract lawyer
	*Documents:*
	Sustainability program for Stockholm Royal Seaport
	Action plan containing municipal sustainability requirements (attached in the land allocation agreements)
	5 formal letters from housing developers to the municipality
	2 responding letters from the municipality
	Minutes from meeting about garages
	*Observations:*
	9 h total (1 general meeting, 1 competence seminar, 1 sustainability forum)
Nature Town (NT)	*Interviews (30–120 min/interviewee)*:
	12 private housing developer project managers (HD NT)
	2 architects working with the housing developers
	2 municipal planning project managers
	*Focus groups:*
	1 with 7 representatives from Luleå municipality (120 min)
	1 with 12 representatives from Luleå municipality (120 min)
	*Documents:*
	Sustainability program for Nature Town
	3 investigations of developers’ perceptions of the municipality
	Mapping of the municipality’s planning process with problem areas

The analysis followed an interpretative and explorative process where the qualitative material was analysed in several steps in order to search for patterns, as opposed to confirming predefined hypotheses (Eisenhardt & Graebner, 2007). The material from each case was first analysed separately by the researcher that had collected that material. This included reading through the interview and focus group transcripts, documents and observation notes in order to familiarise ourselves with the material. Direct interpretations and aggregations of instances from the interviews with the housing developers were then gathered (Stake, 1995). At this stage, any and all challenges related to the housing developers’ implementation of the municipal sustainability requirements that could be identified in the material were compiled, which included both intra- and inter-organizational challenges. These challenges that had been identified in each case were then reviewed by the other researcher, followed by a discussion between the two researchers, addressing any perceived bias through ‘member-checking’ (Yanow, 2014). After this, the direct interpretations and aggregations from the two cases were compared to each other, looking for similarities and differences, in order to assess the consistency of emerging patterns. The various challenges were categorized using a reflexive form of thematic analysis (Braun & Clarke, 2006). This process of abstraction resulted in two main perceived barriers, both of which are closely related to barriers to SC identified in previous research.

After identifying the main perceived barriers, we explored how they are influenced by this particular context, and found that the relationship between developers and municipalities is especially important. To analyse this relationship further, and its influence on the housing developers’ projects, we applied principal-agent theory. We likened the role of the municipalities to that of a principal, since they are setting requirements for the developers in the land allocation agreements, which would subsequently make the developers into agents. We began by using the theory to describe the relationship between the municipalities and developers from this perspective. After this, we consulted the previous literature on principal-agent theory to identify possible implications of this type of relationship, and of different types of contracts, for the agents. These implications were then compared to the developers’ perceived barriers identified in the thematic analysis. This resulted in the identification of aspects of the perceived barriers in this empirical context which could be explained further using principal-agent theory.

## Housing developers’ perceived barriers to implementing municipal sustainability requirements in sustainability-profiled districts

### The relationship between housing developers and municipalities

The municipalities in SRS and NT can be seen as principals since they depend on the housing developers (agents) to develop the new houses in their districts and realise their sustainability objectives. In these cases, the municipalities act as principals by setting requirements in municipal land allocation agreements. The land allocation agreements are outcome-based contracts because they are founded on the housing developers’ achievement of specific requirements. This means that more risk is shifted to the developers as a result of general outcome uncertainty. The municipal sustainability requirements in these agreements go beyond typical land-use regulations: “This is the project with the absolute strictest requirements from the municipality on so many different dimensions” (HD SRS). The municipal sustainability requirements in the land allocation agreements greatly influence the work that is carried out by the housing developers’ project managers during their planning and design phases, preceding their procurement process. During this time they try to implement the municipal sustainability requirements while also producing viable projects that align with their own interests. As agents, the housing developers must weigh the interests of principals with their own self-interest.

“What was in the land allocation agreement has very much governed the way the project looks today, how it has developed. It has mainly been about holding together all of the sustainability requirements with that related to customer value and budget… so we can develop something we are actually able to carry out” (HD SRS)

However, this agency relationship is not entirely clear because the municipalities are technically also suppliers for the housing developers who are buying their serviced building plots. In addition to their monopoly on planning, the municipalities in the two cases own significant portions of land, which makes them important stakeholders for the housing developers who depend on their supply of developable land. The housing developers thereby agree to implement the municipal sustainability requirements from the land allocation agreements in return for receiving the sole right to negotiate buying the serviced building plots in these districts, which also come with a lot of publicity and prestige. There is some confusion over the roles, and consequently the distribution of responsibilities, in this arrangement where “the municipality is after all the owner [principal], but at the same time they [the housing developers] are the ones that carry out the development” (M SRS). The housing developers pay the municipalities for the land and stand for the costs of carrying out their own housing development projects and implementing the municipal sustainability requirements. Much of the costs and risks are thereby placed on the housing developers. The housing developers do not necessarily find this distribution of responsibilities and risks to be favourable or fair.

“Before we started working with this the district got a prize for sustainable urban development… They [the municipality] place a lot of the responsibility and costs on the housing developers for something they are [taking credit for] and I do not think that is a good way to work” (HD SRS)

Regarding the housing developers' relationships with the municipalities, a distinction can be made between the short-term relationship within the temporary organisation that is the housing development project, and the potential long-term relationship. Managing the short-term relationship is essential for delivering the individual project, but the potential long-term relationship they are building with the municipality is of equal importance. This is especially true for housing developers that have not worked with the municipality previously and are using their projects to enter a new market. Housing developers that are already established in the area are also concerned with building up good faith with the municipality given the prospect of receiving favourable land allocations in the future, both within the same sustainability-profiled district and elsewhere. Although it is unclear how this practice is actually carried out, there is an informal understanding that municipalities offer developers more favourable land allocations in the future if they do well in these types of district developments that are considered more difficult. Interviewees from NT refer to this as “förtroendekapital”, which translates to trust capital. It is common for developers to contribute with numerous projects in different stages of the same sustainability-profiled district development. This has particularly been the case in SRS, which started in 2000 and is estimated to take three decades to finish (Sustainability program SRS), while NT started more recently in 2019 (Sustainability program NT).

As a result of some municipal sustainability requirements, the housing developers also have more interdependent relationships with neighbouring developers. For instance, developers in both cases were forced by the municipality to collaborate on the construction of shared facilities, such as garage solutions. Both municipalities have requirements on large underground garages connected between several houses with limited parking for residents and more spaces for bicycles. In their sustainability programs, this is presented as part of an effort to reduce the use of cars, keep car traffic away from the areas around the residential buildings to improve safety and other social sustainability factors, and to promote more sustainable modes of travel, specifically walking, using bicycles and public transport. For the housing developers, this further reduces their autonomy and increases uncertainty and risk in their projects: “The risks here are that we cannot govern by ourselves” (HD SRS).

The housing developers’ principal-agent relationships with the municipalities influence their perceived barriers to implementing the municipal sustainability requirements, presented in the next two sections. Furthermore, issues related to implementing the municipal sustainability requirements are typically resolved through further interaction with the municipalities. These interactions, aimed at resolving the emerging issues related to the perceived barriers, take the form of both formal and informal dialogues and negotiations: “We get a lot of questions and then we need to handle that, create meetings and forums to meet their [the developers’] eventual critique and engage in dialogue” (M SRS). They are carried out in a variety of online (due to covid-19) or in-person meetings between one or several housing developers and the municipalities, as well as workshops, seminars and through emails and formal letters, such as those reviewed in this study.

### Increased financial risk

#### The cost of implementing municipal sustainability requirements

The housing developers stress that implementing the municipal sustainability requirements in SRS and NT “costs a lot” (HD SRS; HD NT). The high cost of implementing many municipal sustainability requirements, combined with high prices on land in the SRS case, means there is less room for other unexpected additional costs in the housing developers’ budgets. In other words, they reduce the developers’ ability to adapt to changes in the economic conditions of their projects, which can be considered as financial flexibility. This entails an important conflict of interest between the housing developers and municipalities. Already being an actor aiming to maximise their economic position, costs of implementing municipal sustainability requirements makes the housing developers more concerned with reducing their costs where they can. The housing developers’ project managers are aware that if they cannot find solutions that meet the requirements set by their own parent organization they will be forced to back out and have wasted the resources and time already put in. The parent organization is another principal with their own interests and requirements, such as budget and time.

“We try to find as cost-effective solutions as possible and that is where the difficulty lies. Everything can be solved but after a while we cannot build it because it will be too expensive” (HD SRS)

The housing developers are fully aware that implementing the municipal sustainability requirements will entail additional costs. These additional costs are first perceived as barriers when unforeseen changes occur. The high complexity in these housing development projects, which is largely a direct result of the many high municipal sustainability requirements, means that there are high levels of uncertainty during the early project phases. The increased uncertainty results in many perceived changes to the housing developers’ understanding of the conditions for their projects, which can make progress difficult: “The conditions are changing all the time and the design for our house has in a way become meaningless” (HD SRS). Flexibility is essential for adapting to these unforeseen changes because “when changes occur there are of course things that cannot be realised and things need to be altered” (HD NT).

#### Need to reduce costs when adapting to unforeseen micro and macro level changes

Increased costs from implementing municipal sustainability requirements become a major barrier when housing developers are forced to adapt to unforeseen changes that constrain their budgets. Unforeseen changes can constrain their budgets either by increasing their costs further or reducing the market value of their final product. The additional costs from the municipal sustainability requirements thereby become more difficult to work into their budgets. However, changes the housing developers consequently wish to make to their designs in order to reduce their costs and meet their new budgets need to be negotiated with and approved by the municipalities, especially if they affect the implementation of the municipal sustainability requirements. The unforeseen changes the housing developers perceive as having the biggest impact on their ability to implement the municipal sustainability requirements include unforeseen technical issues, changes caused by the municipalities and changing market conditions. The first two can be considered as changes caused by micro variables at the project and programme level while the latter changes are the result of macro variables.

*Unforeseen technical issues* related to implementing the municipal sustainability requirements are prominent in both cases and typically emerge at the project level as the housing developers’ work progresses and their understanding of the building conditions increases. The typical issue here is that they realise various solutions will entail more costs to implement than initially expected, for instance “labour costs and alternative costs because this drain chute needs to be included” (HD SRS). Perceived *changes caused by the municipalities,* on the other hand, include alterations made to the municipal requirements and releasing unexpected and important information late in the process. Requirements from the municipalities are subject to modifications during the process leading up to a final development agreement. In the SRS Action Plan there is a whole section listing the “requirements that will be clarified later”. However, even smaller modifications to the municipal requirements can be perceived as big issues for the housing developers that make it difficult for them to predict their costs.

“With these toilets, for example, it said that you ‘can’ and ‘should’ use them, and now it says that we are ‘supposed to’. That is a really big difference. From the municipality’s perspective it is just a way to clarify, but from our, the housing developers’, perspective it means additional costs” (HD SRS).

Housing developers also perceive delays in the release of important information from the municipalities as significant unforeseen changes. This could be interpreted as an instance of asymmetric information where the principal initially has the information advantage, which is not what we would typically expect in a principal-agent relationship. For example, in SRS the municipality conducted their noise investigations after land allocation and in NT the municipality did not carry one out during detailed planning. Meanwhile the housing developers from both cases assumed that this had been done before the land was allocated and they had started working on their projects. This lack of transparency resulted in more uncertainty and wasted resources for the housing developers as they were forced to rethink their designs and material choices.

“We had some ideas that were put on hold with this new noise investigation. We were thinking about buying volume elements from Lithuania but will probably have to reconsider because there will be too many vibrations for that type of construction” (HD SRS)

Finally, *changing market conditions* due to a recent recession greatly affected the housing development projects in both cases. The change in the housing market created much uncertainty and perceived financial risk, making it particularly difficult for the housing developers to form viable project budgets. This macro level change also further exacerbated the issues perceived from the other types of unforeseen changes that require additional costs to adapt to.

“There are probably few budgets that go together today. Nobody is buying for these prices and the land prices do not line up with the actual housing prices that are sinking” (HD SRS)

The unforeseen changes presented above, particularly the changing market conditions, made all of the housing developers in both cases focus more on exploring options for reducing their costs. Since the municipal sustainability requirements entail increased costs for the developers, attempting to remove or alter some of them through negotiations with the municipalities becomes one option that they explore: “I think everyone has been working towards not having to implement this solution” (HD SRS). Modifications can also be made to other types of municipal requirements to make implementing more of the sustainability requirements possible. For instance, in order to adapt to the new market several housing developers in NT were allowed to change their projects from building condominiums to rentals, which is regulated in the detailed development plans. However, this transition to rental apartments meant that the developers had to reconsider many other aspects of their projects. Since the transition was done quite late in the planning and design process this also entailed wasted resources for the developers. Furthermore, other changes proved to be difficult to make given the context of their land allocation agreement with the municipality.

“When the situation only got worse and worse a decision was made to switch to rental apartments. However, the municipality’s requirement was that we should build an underground garage under the house. We were not allowed to change the garage which made this very expensive.” (HD NT)

An issue that makes adapting to these unforeseen changes more difficult for the developers is working with too many details too early in the process as result of having a lot of specific municipal sustainability requirements in land allocation agreements. This is problematic since resources are put into developing aspects that will likely need to be altered as the projects progresses and the conditions imposed by the project environment change.

“As a developer you do not want things to be too detailed too early because it can be many years before construction starts and then the market and technology can change… it has been too detailed because we developed our solution a couple of years ago and the market has changed and the customer category we saw then we might not have today” (HD NT).

Reducing asymmetric information through good communication with the municipality is essential for implementing municipal sustainability requirements when adapting to unforeseen change. Since changes need to be negotiated with the municipalities, it is important that the municipalities understand why they are necessary from the housing developers’ perspectives, especially since the housing developers have more information and knowledge regarding the practicalities of actually implementing the municipal sustainability requirements in their projects.

“There is some kind of constant change in the conditions. Then you cannot assume that things are set in stone, but you have an ongoing dialogue about why you make changes, if you make changes, and ensure that both parties think this is the best solution” (HD NT)

### Conflicting objectives

#### Actors’ conflicting interests result in conflicting objectives and requirements

Conflicting interests between developers and municipalities are perceived as a major barrier when these interdependent actors are unable to align these interests and find implementable solutions that they can agree on. The housing developers and municipalities have different interests and objectives from each other, which become especially apparent in the face of unforeseen changes such as those discussed previously. For example, the housing developers are sensitive to changes in the market, which is not necessarily the same case for the municipalities who focus more on the long-term urban development in their cities, as illustrated by the following quote.

“You want to deliver what all stakeholders are demanding at the time when you go out with a product, what the market needs or what the municipality thinks they need, because that can change over time. Maybe there is a boom when this product comes out and everyone will be building condominiums, then there is a recession and everyone will build small rental apartments, but maybe the municipality still wants something else to be built.” (HD NT)

The municipalities in the two cases want to drive sustainable innovation by challenging the housing developers with many difficult requirements in their land allocation agreements. Since these are outcome-based contracts, this means that the housing developers end up taking on a lot of risk. While the housing developers that take part in these sustainability-profiled district developments generally want to contribute to sustainable development and innovation as well, they also want to reduce uncertainty and risk in their projects. This interest in reducing uncertainty and risk is antithetical to driving innovation.

“Investigating new materials and construction techniques is something we would rather not do because we want to use proven methods” (HD NT).

It is also clear that the housing developers do not always agree with the particular issues and solutions that the municipalities have chosen to prioritise. While the municipalities naturally want the developers to meet as many of their sustainability requirements as possible, implementing them is sometimes in conflict with the housing developers’ own self-interests. For instance, implementing the municipal sustainability requirements in the two cases is resource intensive and thereby entails high costs for the housing developers, as discussed previously. This goes against the interest of the housing developers who are generally concerned about keeping their costs down as this affects both their budget and potential future profit. For this reason, the developers in both cases question whether each municipal sustainability requirement is worth the additional costs and try to oppose requirements they do not consider worthwhile.

“Is this sustainable in the sense that you can motivate the costs for it?... The cost for implementing this is very high... and at the same time the benefits are limited” (HD SRS).

The housing developers are concerned about how different solutions will impact the value of their final product and what they expect that their buyers will want. Municipal sustainability requirements that are considered to reduce the value of their final product, either by being undesirable for potential buyers or having costs that exceed potential benefits, are in conflict with the housing developers’ interests. For this reason, the developers will generally be opposed to implementing these requirements.

“An enormous responsibility is placed on our buyers. I am not sure that our buyers are interested in this solution. I think it will be more difficult to sell the apartment. And above all, it poses a huge risk to the buyers and the association that will run this facility.” (HD SRS)

On the other hand, the developers will prioritise municipal sustainability requirements that they believe will increase the value of their product. The developers are after all motivated to meet the objectives and interests of their principals, as well as their own interests. For this reason, they will be especially driven to meet these requirements.

“Everything that you can connect to customer value we see as more beneficial. It could be things like the shared facilities, a greenhouse in the courtyard, or solar cells on the roof so that the residents feel like they live in a modern building.” (HD NT)

In addition to weighing the potential benefits and costs of various requirements, housing developers are also more concerned with the feasibility of actually implementing them in their projects. During their planning and design processes it was discovered that several of the municipal sustainability requirements in the two cases are in direct conflict with each other. This would not have been as obvious for the municipalities that are less concerned with the practicalities of actually implementing them. Implementing all of the municipal sustainability requirements in the same housing development project is thereby perceived as technically impossible.

“Certain requirements in the detailed development plan are not technically feasible. For example...the closed neighbourhoods and the daylight requirement in Miljöbyggnad [a Swedish environmental certification] are not compatible… You do not notice this until you start looking at details, which is a shame because it costs quite a lot to do a competition... A lot of requirements have been set without knowing what they entail in practice when you start building” (HD NT)

Some municipal sustainability requirements were even discovered to be in direct conflict with national building regulations and thereby not legally feasible to implement, alluding to conflicting interests between local and national authorities.

”They say we should have green roofs and a lot of vegetation on the roofs, but at the same time it is not approved from a fire safety standpoint... So the sustainability program requires us to build something that we are actually not allowed to build” (HD SRS).

As mentioned previously, some requirements from the land allocation agreements force the housing developers to collaborate over shared facilities. This means that the municipalities are forcing them to collaborate with their future competitors. While the housing developers are interdependent, their relationship is not hierarchical and typically not contractual either, meaning they do not consider the other housing developers as principals. This means that the housing developers will consider their own self-interest and the interest of their principals, such as the municipalities, more important than the interests of the other housing developers. When too many of the housing developers are forced to collaborate, conflicting interests make it particularly difficult for them to agree on one solution. While the housing developers do have different interests and priorities, conflicting interests between them are largely rooted in their similarities. They typically expect the other housing developers to act opportunistically and do everything they can to maximise their own economic position because that is what they themselves intend to do.

“There is not one developer who does not optimize his own situation because this is about so much money. That can be the difference between getting a project together and not getting a project together… No one gives up anything voluntarily to make it better for others and worse for themselves. This results in either not agreeing, meaning you cannot move forward, or the issue is buried until you are forced to address it” (HD SRS)

#### Addressing conflicts when implementing municipal sustainability requirements

Housing developers try to resolve issues that emerge as a result of conflicting interests and requirements through dialogue and negotiation with the municipality. As the principal, the municipality has the last say regarding modifications to, or the complete removal of, municipal sustainability requirements. On the other hand, as the agents the housing developers typically have the upper hand with regards to knowledge and information about the technical and practical aspects of implementing the municipal sustainability requirements in their projects. This is partly because they are in the business of carrying out housing development projects and thereby have previous experience to draw on, and because they are carrying out these housing development projects and put a lot of work into trying to implement the requirements in their designs and planning. For this reason the housing developers are often able to make their case which typically enables them to get changes through when they are deemed vital.

“It is probably no problem to meet these requirements if you have a little freer reins somewhere. But now I have solved it. We have been to the municipality where we had an architect with us to justify why we had to divide it into three houses and prove with daylight, and other things, that we could not solve it in any other way... We have spent a lot of time investigating, changing and proving to the municipality why it is not possible to do this… We meet the requirements in the detailed plan and Miljöbyggnad, but we do not follow the competition proposal to the letter. We have tried as far as we can" (HD NT)

How well inter-organisational conflicts are managed and whether or not the actors are able to find solutions to problems largely depends on their communication with each other. The frequent emergence of different types of conflicts is seen in both cases, although the gravity of the problems can vary significantly. This makes it important for actors to have good communication with each other and transparency for building trust. Since the housing developers depend on the municipalities to keep them correctly informed about many aspects, there are several instances where the principal has the information advantage, which is less commonly discussed in relation to principal-agent relationships. The developers generally consider their communication with the municipalities to be good. However, there are instances where there is a lack of transparency and communication between the actors which hinders conflict resolution. A lack of communication directed at reducing asymmetric information and knowledge prevents the actors from aligning their interests and objectives.

“We thought that the question regarding noise was evaluated when we got the land allocation agreement. It turns out that it was not. We feel that they allocated land too early and were not aware of the conditions. We got incorrect and poorly supported analyses concerning their ambitions… We cannot plan… And we have not gotten any feedback on how the sewer system and garage will work either. So we are not doing anything.” (HD SRS)

When communication from the municipality is slow, one consequence is that “there are a lot of rumours” (HD SRS), causing confusion and suspicion amongst the housing developers. Another common issue is that the people from both the municipality and housing developers are sometimes swapped out. It can therefore be unclear for the developers whom they should be talking to and through what channels, which is further complicated when communication within the municipality is lacking.

“There has been a dialogue and in the end we have agreed on what we should do. Then it turns out the agreements that have been made with [specific municipal project managers] may not apply now when we have come to the building permit. Then they have a completely different view of the matter... They have apparently not talked to each other" (HD NT)

A lack of information and knowledge about the land allocation and development process also caused issues for the developers in both cases. For example, there is much uncertainty over which municipal sustainability requirements are negotiable, meaning the developers might be investigating possible modifications to requirements to align their interest better that the municipality is not actually able to make modifications to. A lack of information regarding how the developers were chosen by the municipality also resulted in several developers perceiving the land allocation process as unfair. This created friction between the housing developers.

“Some who won have promised a lot. They have not thought it through or they do not care and accept penalties... To win, you had to say how far you would go down in energy usage and those who won got to choose blocks first, but can they actually follow through?” (HD SRS)

The importance for the housing developers to build and maintain a good relationship with the municipalities, which is one of the main drivers for them to build in these district developments, sometimes hindered communication. The prospect of generating trust capital for more favourable land allocations in the future led housing developers to self-censor, exacerbating asymmetric information between them and the municipalities and creating a stark contrast between the challenges expressed during seminars and workshops and those discussed in interviews. They are reluctant to openly oppose requirements from the municipality unless the other developers follow suit. If several housing developers perceive similar issues with certain municipal sustainability requirements they are more likely to address them, because if they can approach the municipality as a group they are less likely to be singled out and labelled as being difficult to work with. In SRS, the housing developers created a forum on their own initiative so that they can raise concerns to the municipality together. This was done by drafting formal letters to the municipality, such as those reviewed in this study, which listed all of the housing developers’ current concerns over specific municipal sustainability requirements and were then signed by several of the developers.

”It is always sensitive to be the one that is troublesome towards the municipality. We have a long-term relationships with the municipality and then you do not want to be the only one that causes difficulties when everyone else follows” (HD SRS).

A lack of communication and transparency between actors generally results in significant adverse effects that can be seen in both cases, namely distrust between actors, confusion over the division of responsibilities, the spreading of rumours and simply postponing solving problems for later in the process. These effects ultimately result in higher perceived uncertainty for the housing developers which may lead to additional perceived changes. A lack of transparency can lead to distrust between actors if they lack knowledge and understanding of each other’s intentions, motives and abilities. For example, some of the housing developers do not expect the municipality to act in their best interest, whether intentionally or unintentionally.

“As I see it, the market is saturated with new builds. And I think the municipality will want to release too much too quickly.” (HD SRS)

Municipalities and developers are different meaning more is required from them to build up a good understanding of each other. For this reason, the municipalities are just as likely not to trust the housing developers. Some interviewees from the municipalities in both cases expressed distrust and apprehension regarding the housing developers’ perceived barriers to implementing their requirements. They suspected that their complaints were unfounded and exaggerated and sought to protect themselves from this moral hazard. Housing developers from NT also perceive the high degree of detail in the detailed development plan and land allocation agreements as a form of micromanaging, which typically indicates a lack of trust.

“I reacted the most to this thing with the facade material that was decided from the beginning… There are lots of things that we could have solved to everyone's satisfaction at a later time. They do not trust us, and not only us, but everyone else as well” (HD NT)

Good communication is also needed between the housing developers to avoid ambiguity and confusion over the division of roles and responsibilities and to reduce uncertainty. However, when conflicts emerge between the housing developers that they struggled to resolve on their own they expect the municipalities to intervene and take an active role in helping them, which they are not necessarily inclined to do.

“We are building a garage together so when they do not start their project we are left without any parking spaces. But even when they start their projects, how should we divide the costs? Who will be responsible for the project management? Who is the owner?... We could also build for the municipality and make them the owners, but they did not want to do that... They wanted us to build it together which creates a lot of anxiety and uncertainty among the developers” (HD NT)

If problems remain unresolved it is common that they are simply pushed forward to be resolved later in the process, for instance through “dialogue along with the building permits” (HD NT). However, this is more likely to create bottlenecks that further impede their implementation of the municipal sustainability requirements.

## Discussion

The findings illustrate how developers’ perceived barriers to SC, here categorised into increased financial risk and conflicting objectives (c.f. Opoku & Ahmed, 2014; Osmani & O'Reilly, 2009; Shen et al., 2017; Williams & Dair, 2007; Zainul Abidin et al., 2013), are also perceived as barriers when implementing municipal sustainability requirements in sustainability-profiled districts. To answer the first research question, we elaborate further on how these barriers emerge and are perceived during the implementation process, which poses its own set of challenges (c.f. Harty, 2008), and the particular implications that they have for housing developers in the context of flagship projects with high ambitions on sustainability. Regarding increased financial risk, the in-depth exploration revealed that the housing developers first perceive this as a major barrier when they need to adapt to unforeseen changes that constrain their budgets. In SRS and NT, this included unforeseen technical issues and changes caused by the municipality, which typically increased the housing developers’ costs, and the downturn in the housing market which decreased the expected value of their final product. High costs for implementing municipal sustainability requirements reduce the housing developers’ ability to fit other project costs into their budgets (c.f. Storbjörk et al., 2018). Unforeseen changes that constrain their budgets therefore result in some municipal sustainability requirements being perceived as too risky regarding costs and value for money (c.f. Häkkinen & Belloni, 2011; Ivory, 2005). Similarly, a perceived lack of customer demand for municipal sustainability requirements that entail innovation becomes a concern for housing developers who are driven by the market (Hagbert & Femenias, 2016; Storbjörk et al., 2018). The unexpected constraints on their budgets make them more risk averse and less willing to invest in innovations that they consider to show little promise for increasing the value of their product (Häkkinen & Belloni, 2011; Zainul Abidin et al., 2013).

Previous research has identified conflicting objectives as a major barrier to implementing SC (e.g. Francart et al., 2019; Williams & Dair, 2007). Here we see that conflicting objectives, in the context of housing development in sustainability-profiled districts, are largely rooted in conflicting short-term and long-term interests between the housing developers and municipalities. This is in addition to the typical conflicts between different types of sustainable urban development goals (Martin et al., 2018). The housing developers in both cases are developing condominiums that they will sell after completion, although some developers in NT changed to rental apartments when the market declined to align their interests more with the municipalities’ long-term interests. As a result, they generally have a short-term focus on costs and productivity within the individual project (c.f. Häkkinen & Belloni, 2011; Vennström & Eriksson, 2010). It was also observed that they became increasingly concerned with the technical feasibility of implementing various solutions as their projects progressed. The municipalities, on the other hand, are more interested in the long-term sustainable development in their cities. Weighing different interests from principals with one’s own self-interests and being forced to find compromises and trade-offs between them is characteristic of the agent role in principal-agent relationships (Waterman & Meier, 1998). These differing interests and concerns, which are often in conflict with each other, lead to a variety of conflicting objectives and requirements. For instance, there are several instances where the municipal sustainability requirements are in direct conflict with each other regarding technical properties, meaning it is not possible for the housing developers to find solutions that satisfy all of them, which Williams and Dair (2007) found in their study as well. The conflicts between driving sustainable development and the housing developers’ other objectives are in this context not internal for the developers since it is the municipalities that are pushing for the sustainable development and innovation.

The effects of the barriers explored here are exacerbated by each other and there is some notable overlap between them. Given the emphasis on increased costs and a lack of benefits in previous literature on developers’ perceived barriers to SC (e.g. Opoku & Ahmed, 2014; Osmani & O'Reilly, 2009; Shen et al., 2017; Williams & Dair, 2007; Zainul Abidin et al., 2013), and the distinct and significant implications observed in the case studies, we settled on categorising financial risk as a specific barrier. However, conflicting interests between the municipalities’ long-term objectives and the housing developers’ short-term objectives are arguably the main underlying issue for both sets of barriers presented here. In this empirical context, financial risk and conflicting objectives should therefore be considered as distinct and notable barriers that are both rooted in conflicting interests between housing developers and municipalities, rather than being completely separate from each other. Applying principal-agent theory here highlights how a major challenge for the housing developers is weighing these conflicting interests and finding compromises and trade-offs between them, while the municipalities, in their role as principals, can try to find ways to align them through the use of incentives (c.f. Shapiro, 2005; Waterman & Meier, 1998). On the flipside of this, given the principal-agent relationship between the housing developers and municipalities, the housing developers are extra motivated to work on solutions that align with both their own and the municipalities’ interests and objectives.

Previous literature has generally assumed that developers act as principals in relation to driving and implementing SC (c.f. e.g. Häkkinen & Belloni, 2011; Isaksson & Linderoth, 2018) and innovation more generally (c.f. e.g. Havenvid et al., 2016; Kulatunga et al., 2011). Here we illustrate how, in the context of developers’ relationships with municipalities in Swedish sustainability-profiled districts, municipalities are instead adopting the role of the principal using outcome-based contracts and the developers are in turn acting as agents (c.f. Eisenhardt, 1989a; Turner & Müller, 2004). This supports Hagbert and Malmqvist’s (2019) claim that contemporary sustainable housing development practices are changing the roles of public and private actors. However, we can also deduce that this is not an entirely typical principal-agent relationship. For instance, the developers do not always have the information advantage, as previous literature on principal-agent theory would suggest should be the case (c.f. Eisenhardt, 1989a; Jensen et al., 2006). Furthermore, although the municipalities place requirements on the developers, they can also be seen as the developers’ suppliers of buildable land. Either way, the process of implementing the municipal sustainability requirements in the housing developers’ projects involves complex negotiations between multiple actors, as is generally the case for innovation in construction (Harty, 2008). However, since new solutions are imposed on these housing development projects by municipalities, we illustrate how negotiations over emerging issues related to their implementation are first held between the housing developers and the municipalities in the district, as opposed to other actors within the construction projects, answering the second research question.

In housing development projects in sustainability-profiled districts, early decisions on solutions and designs are largely dependent on the land allocation agreements and the relationship with the municipality, as Caesar (2016) has previously pointed out is the case for most housing developments on municipal land. For this reason, good communication between these actors, including a clear division of responsibilities, is vital for resolving emerging issues and reconciling conflicting interests (c.f. Taylor et al., 2012; Turner & Müller, 2004). Findings also strengthen Caesar’s (2016) conclusion that housing developers are highly dependent on the supply of suitable land and this strongly dictates their need to develop good relationships with municipalities in Sweden that are big landowners, which is sometimes even pursued at the detriment of the project at hand. The findings illustrate how the work from land allocation to a final development agreement is a complex and ‘fuzzy’ process that neither the housing developers nor the municipality have full visibility or control over. During the front-end, the housing developers in these district developments are ultimately seeking to increase their flexibility when adapting to unforeseen changes, by for example reducing their costs and negotiating modifications to the municipal sustainability requirements, and simultaneously reduce uncertainty and risk (c.f. Storbjörk et al., 2018).

## Conclusions

### Theoretical contributions

The purpose of this study is to explore how housing developers’ perceived barriers to SC influence their implementation of municipal sustainability requirements, and to contextualise this within their relationship with municipalities in Swedish sustainability-profiled districts. Developers perceived barriers to SC are categorised into increased financial risk and conflicting objectives and explored in two sustainability-profiled district developments in Sweden. The findings illustrate how increased financial risk caused by high costs of implementing municipal sustainability requirements is first perceived as a major barrier when housing developers are faced with unforeseen changes that constrain their project budgets. Conflicting objectives are largely rooted in conflicting short-term and long-term interests between the housing developers and municipalities. The principal-agent relationship dynamics between the housing developers and the municipalities ultimately shape the housing developers’ perceptions of, and possibilities for, addressing these barriers. The findings contribute to the growing discussion on the perspective of property developers and their role in sustainable urban development (e.g. Taylor et al., 2012; Hagbert & Femenias, 2016; Storbjörk et al., 2018; Hagbert & Malmqvist, 2019; Brokking et al., 2020; Kågström, 2020). Contributions are also made to literature on the use of municipal sustainability requirements in public land development (e.g. Bulkeley & Kern, 2006; Francart et al., 2019; Smedby, 2016; Smedby & Quitzau, 2016; Tambach & Visscher, 2012) by exploring the implications from the perspective of the housing developers and applying principal-agent theory to their relationship with municipalities. Finally, contributions are made to the general knowledge of developers’ perceived barriers to SC (e.g. Deng & Wu, 2014; Häkkinen & Belloni, 2011; Isaksson & Linderoth, 2018; Zainul Abidin et al., 2013) by presenting a distinct empirical context where they are forced to implement new and challenging project-specific requirements set by municipalities.

### Policy implications

The findings have implications for municipalities that want to use land allocations to drive sustainable urban development by challenging private sector actors to implement sustainable innovations in their projects. While municipalities are able to drive sustainable development in projects taking place on municipal land, they cannot ensure that their influence extends beyond these individual projects. However, flagship demonstration projects offer the potential of changing mainstream construction practices through the dissemination of new knowledge, which we would argue must be co-created by the municipalities and developers together. An important element in developing these new solutions and practices that the construction industry might actually be willing to adopt en masse lies in the developers’ ability to find innovative ways to make them feasible and profitable. Exploring how various requirements affect housing developers and their projects reveals the extent of the municipalities’ influence and their limitations. For example, municipalities’ ability to produce viable opportunities for housing development projects with high requirements on sustainability is heavily dependent on market conditions. Municipal sustainability requirements result in additional costs for the housing developers, so when the market is stagnant the housing developers struggle to form their budgets. For these reasons, it is not enough for municipalities to govern housing development projects in an authoritative manner, but they should also find ways to enable more flexible and collaborative forms of problem solving.

Since municipalities are important principals for housing developers, they are motivated to find solutions that align with both actors’ interests and objectives. According to principal-agent theory, the challenge lies in aligning them (c.f. Eisenhardt, 1989a; Hart & Holmström, 1987; Waterman & Meier, 1998). The study further supports suggestions previously made by Francart et al. (2019), namely that municipalities should work on establishing and maintaining good communication with developers to help align their objectives and reduce misunderstandings regarding responsibilities. Since conflicting interests between actors result in conflicting requirements, it would also be beneficial for the actors to have a better understanding of the others’ perspectives. Municipalities should take more time to communicate their own interests and perspective to the housing developers and try to gain a better understanding of the housing developers’ interests and perspectives. This would improve their ability to find desirable solutions to conflicting requirements in their dialogues with developers, and could perhaps help them avoid major issues in future projects, or at least recognise earlier on which requirements might cause problems.

### Practical implications for housing developers

The findings also have practical implications for housing developers implementing municipal sustainability requirements in sustainability-profiled districts. They provide indications of the obstacles that housing developers’ project managers will face when they are forced to implement municipal sustainability requirements. Creative problem-solving is sometimes required when implementing challenging municipal sustainability requirements designed to drive innovation. This can entail exploring ideas that do not come to fruition, which requires both time and resources resulting in higher costs and financial risk for the housing developers. The planning and design phases of housing development projects in these types of sustainability-profiled district developments can therefore be expected to take longer and cost more than typical projects would.

### Limitations and suggestions for future research

The municipalities studied here use land allocation agreements with high requirements on sustainability to create a set of conditions that the private housing developers must work with during the early planning phases of their projects. This will influence their solutions and consequently their procurement process, making the municipalities important change agents and principals for the developers. The housing developers' intermediary role, where they are both being governed by municipal sustainability requirements and governing their projects through procurement, in other words acting as both an agent and a principal, is thus in need of further investigation. For example, possible questions for future research could be how developers translate, or materialize, municipalities’ sustainability requirements when designing their procurement strategy. Another question for future research is procedures for following up on sustainability requirements. Are the sustainability requirements met on a short and long-term basis, and if not, why?

The findings indicate that housing developers play an active role in determining the ways in which municipal sustainability requirements are implemented into their projects. The requirements established during this phase will determine the rest of their project, along with the planning and relationship building that takes place. Future research could therefore investigate the housing developers’ role in governing the process of implementing municipal sustainability requirements further in other parts of their projects. The focus in this paper is also on housing developers’ contributions to implementing SC in their projects. However, they may play important roles in contributing to sustainable development goals by addressing other issues in housing development and transforming the industry in other ways. Developers’ roles in addressing other important aspects of sustainable housing development should be explored further.

Here we illustrate how conflicting interests between housing developers and municipalities initially make the process of problem solving in housing development projects more complex. Given their importance during the transition from land use planning to housing development, the interests of these two actors should be explored further to develop more knowledge of how and when they are aligned and in conflict. Here we also allude to the collaborative problem solving process that takes place between housing developers and municipalities to resolve issues related to implementing municipal sustainability requirements, but do not focus on delving into the actual process. Therefore, we call for more research that explores this collaborative problem solving process between housing developers and municipalities. In addition to this, the findings suggest that collaboration between developers in district development stages play an important role in resolving issues related to implementing municipal sustainability requirements, which could be explored further.

Here we have explored the use of municipal sustainability requirements in land allocation agreements in two Swedish municipalities. However, these practices are very specific to national and even city contexts. For this reason future research should also explore this practice further in other contexts to improve generalizability. Future studies could look at different types of urban developments carried out by municipalities of different sizes and with different resources. This study also focused on perceived barriers and did not lay much emphasis on enablers. Future studies could therefore explore perceived enablers in this empirical context.

## Data Availability

The material analysed during the current study is available from the corresponding author on reasonable request.
